# The SMAC mimetic AT-101 exhibits anti-tumor and anti-metastasis activity in lung adenocarcinoma cells by the IAPs/ caspase-dependent apoptosis and p65-NFƙB cross-talk 

**DOI:** 10.22038/ijbms.2021.56400.12586

**Published:** 2021-07

**Authors:** Irfan Ahmad, Safia Irfan, Mirza Masroor Ali Beg, Hossam Kamli, Syed Parveen Ali, Naseem Begum, Mohammad Y Alshahrani, Prasanna Rajagopalan

**Affiliations:** 1 Department of Clinical Laboratory Sciences, College of Applied Medical Sciences, King Khalid University, Abha, Saudi Arabia; 2 Department of Physiology, College of Medicine, King Khalid University, Abha, Saudi Arabia; 3 Department of Biochemistry, Maulana Azad Medical College, New Delhi, India; 4 Faculty of Medicine, Ala-Too International University, Bishkek, Kyrgyzstan; 5 Centre for Promotion of Medical Research, Ala-Too International University, Bishkek, Kyrgyzstan; 6 Central Research Laboratory, College of Applied Medical Sciences, King Khalid University, Abha, Saudi Arabia

**Keywords:** AT-101, Caspases, IAPs, NCI-H522, NFƙB, SMAC mimetic

## Abstract

**Objective(s):**

The Inhibitors of Apoptosis (IAPs) regulate initiator and effector phases of caspase mediated apoptosis. This study evaluates the effects of SMAC mimetic AT-101 in regulation of IAPs/caspases/NFƙB-p65 in an adenocarcinoma cell line.

**Materials and Methods:**

MTT assay was performed in the NCI-H522 cell line. Flow cytometry was used for detecting cell cycle, apoptosis, and NFƙB-p65 regulation. Effects of AT-101 on IAPs and caspases were determined by quantitative real time-PCR and western blotting. AutoDock-VINA was used for computational analysis.

**Results:**

AT-101 reduced the cell proliferation of NCI-H522 with a GI50 value of 7 μM. The compound arrested adenocarcinoma cells in the G1 phase of the cell cycle and increased early and late phase apoptosis while decreasing tumor-cell trans-migration. AT-101 treatment to NCI H522 at a concentration of 0.35 μM decreased XIAP, cIAP-1, and cIAP-2 mRNA levels to 4.39±0.66, 1.93±0.26, and 2.20±0.24 folds, respectively. Increased dose of AT-101 at 0.7 μM concentration further decreased XIAP, cIAP-1, and cIAP-2 mRNA levels to 2.44±0.67, 1.46±0.93, and 0.97±0.10 folds, respectively. Similar effects of a dose-dependent decrease in the protein expressions of XIAP, cIAP-1, and cIAP-2 were observed with AT-101 treatments, while a dose-responsive increase in the mRNA and protein expression levels of caspase 6 and caspase 7 was observed in the NCI-H522 cell line. The compound exhibited binding affinity (-6.1 kcal/mol) and inhibited NFƙB-p65 in these cells.

**Conclusion:**

AT-101 had anti-tumor efficacy against lung adenocarcinoma cells which could be mediated through IAPs/caspase-dependent apoptosis and NFƙB-p65 cross talk. Results from this study suggests a signal cross talk between IAPs and NFkB and open new channels for further investigations in therapeutic intervention against lung cancer management.

## Introduction

Lung cancer is one of the prominent causes of mortality due to cancer. Non-small cell lung cancer (NSCLC) covers 75–80% of almost all lung cancer types (1). In spite of noteworthy advances in medication and surgery, lung cancer prognosis is still poor. Adaptive resistance frequently leads to temporary responses and subsequent progression of the disease, while the survival rate in NSCLC patients is only 15% (2). Programmed cell death comprises apoptosis, autophagy, and necrosis (3). The apoptosis mechanism plays a key role in removing the cancer cells and results in the suppression of the tumor (4). The IAPs (inhibitor of apoptosis proteins) family incorporates a number of inconsistent proteins that were originally delineated as inhibitors of apoptosis and hardly any of them were involved in the neutralization of caspases. IAPs share the baculovirus IAP repeat domain, which performs as an E3-ubiquitin ligase to additionally apply a number of auxiliary functions in non-apoptotic pathways that are known to be involved in invasion, migration, and metastasis (5). Studies reveal that XIAP could inhibit caspase-3 and -7 using evolutionarily conserved binding sites (6). It is also shown that XIAP could interact potentially with caspase-9 at its BIR3 domain, while XIAP interaction with caspase-3 and -7 happens at its BIR2 domain, thereby interfering with both death signaling pathways (7). On the other hand, evidence indicates that cIAP1 and cIAP2 (Cellular Inhibitor of Apoptosis Protein 1 and Cellular Inhibitor of Apoptosis Protein 1), may inhibit caspase activity and apoptosis in a more indirect manner particularly at the level of death receptor signaling. Reports show that both cIAP1 and cIAP2 regulate TNFα-mediated NFƙB (Nuclear factor-kappa B) activation to execute apoptosis via TRAF2 (Tumor necrosis factor (TNF) receptor-associated factor-2) in cancer cells (8, 9). 

XIAP is atypically expressed in many human cancers and has been reported to cause drug resistance in the chemotherapy regimen. Increased expression of XIAP in ovarian cancer cells showed chemoresistance after cisplatin treatment (10). Therapy response is diminished in AML (acute myeloid leukemia) patients with frequently overexpressed XIAP mRNA and protein levels (11). This has resulted in reciprocation of routine treatment with combined chemotherapy and high-dose radiation therapy for AML treatment (11). Moreover, increased levels of IAPs have also been revealed for ovarian cancer (10), B-cell Hodgkin, Non-Hodgkin lymphoma (12), renal cancer (13), esophageal cancer (14), and NSCLC (15). Expression of XIAP is also considered a prognostic marker since it corresponds with tumor combativeness and progression in renal cancer (16). IAPs constitute feasible targets for explicitly neutralizing resistance mechanisms therapy causing relapse and therapy failure. XIAP inhibits the cell death effects by causing their proteasomal degradation and physical interaction with caspases (17, 18). In another way, it also activates survival signaling through the NFkB pathway thereby shifting the balance of survival and death in tumor cells towards NFkB-driven survival and death resistance. 

The second mitochondrial-derived activator of caspase (SMAC) as a negative regulator of XIAP, can enhance apoptosis by binding to XIAP and preventing it from binding to caspases (19). As numerous SMAC-mimetics have been exhibited to deactivate circulating cancerous cells, such drugs may be useful in clinical applications of cancer treatment and metastasis (17). Numerous SMAC-mimetics have been reported to exhibit effective anticancer activities in preclinical findings. These include monovalent SMAC mimetics or small biochemical compounds, which inhibit the BIR domains of XIAP and other IAPs to sensitize or stimulate apoptosis (20). AT-101 (R-(−)-gossypol acetic acid), a pan Bcl-2 inhibitor, has revealed anticancer activity in some cancer cell lines (21). Therefore, the current study aimed to evaluate the role of AT-101 in the expressions of IAPs, caspases, and NFƙB in the NCI-H522 lung cancer cell line.

## Materials and Methods

***Materials***

All reagents, chemicals, and AT-101 compound were obtained from Sigma-Aldrich (St. Louis, MO, USA). NCI-H522 and HUVEC cell lines were obtained from the American Type Culture Collection (ATCC). Flow cytometry kits, migration kit, and primary antibodies were from Millipore, Thermo Fischer Scientific Corp, CA, USA. Western blot antibodies were purchased from Abcam (Cambridge, UK). RNA was isolated through all untreated and treated cell lines using Trizol (Invitrogen) reagent. Reverse transcription and RT-qPCR reactions were carried out by SYBR Green I technology (Integrated DNA Technologies, Coralville, Iowa, USA). 

***Methods***

*Cell culture*

NCI-H522 cells were cultured in RPMI-1640 media supplemented with 0.01 mg/ml of insulin, 10% of fetal bovine serum (FBS), 100 μg/ml of streptomycin, 100 U/ml of penicillin, 250 μg/ml of gentamicin, and 250 ng/ml of amphotericin. HUVEC cells were maintained in F­12K growth medium (10% FBS, 100 U/ml of penicillin, 250 μg/ml gentamicin, 250 ng/ml of amphotericin) supplemented with 0.1 mg/ml heparin and 0.05 mg/ml endothelial cell growth supplement (ECGS). Cells were maintained at 37 °C in a 5% CO_2_ hatchery containing sticky air. The medium was replaced every alternative day and the maintenance was sternly followed in accordance to the manufacturer. Assays were accomplished when the cells were ≤70% confluent. 

*Cell viability assay*

MTT assay was accomplished to calculate the GI_50 _(Growth inhibitory 50) value for SMAC mimetic compound AT-101 (Sigma-Aldrich, USA) (22). Briefly, 5 X 10^3^ cells/well in the usual growth media were incubated for 24 hr and different concentrations of AT-101 were added along with a DMSO blank. The cells were incubated at 37 °C and 5% CO_2 _for 72 hr. MTT at a concentration of 5 mg/ml in 25 µl was added and incubated for four hours. After aspirating the media from the wells, the formazan products were dissolved in 250 µl of sterile DMSO. The color was read at 560 nm for absorbance; 640 nm was used as the reference wavelength. The day 0 values were subtracted and the reduction in the percent of cell viability was analyzed to determine the GI_50_ value. 

*Cell cycle assay*

NCI-H522 cells were treated with 3.5 µM or 7.0 µM AT-101 for 24 hr with suitable blank and incubated at 37 °C in a CO_2_ incubator. Cells were briefly washed with sterile PBS and fixed with 70% ethanol until analysis. Cell cycle analysis was carried out using a kit from Millipore (Cat. No. FCCH025142) and 10,000 events were acquired on a Guava easyCyte™ flow cytometer. Express Pro software from Millipore was used for the analysis. 

*Apoptosis assay*

Annexin V assay kit from Thermo Fischer Scientific was utilized to determine apoptosis in NCI-H522 cells. 0.5 × 10^6 ^cells were treated at 3.5 µM or 7.0 µM AT-101 for 48 hr followed by incubation in a CO_2 _incubator. After staining with 0.30 µg/ml Annexin V reagent for 15 min in the dark, the cells were briefly washed twice with PBS and re-suspended in the same solution which had 0.5 µg/ml propidium iodide. 10,000 events were acquired in a Guava easyCyte™ flow cytometer and the data analysis was performed using InCyte software from Millipore Corp, USA.

*Tumor cell trans-endothelial migration analysis*

The assay was performed with a QCM™ Tumor Cell Trans-Endothelial Migration Assay –kit from Millipore as per the manufacturer’s instructions. 1.0 X 10^5 ^HUVEC cells were cultured on the provided migration inserts till they grew confluent. After removing the media from inserts, an equal number of NCI-H522 cells that were starved overnight in FBS free media was added to the inserts. These inserts were moved to new wells that contained serum-free growth media with or without 25 ng/ml HGF (hepatocyte growth factor) at the final concentration. The tumor cells were allowed to migrate across the membrane for 12 hr in a CO_2_ incubator. After which the media was removed, stained, eluted. and transferred to a 96-wells plate to read absorbance at 540 – 570 nm. The intensity of the color produced was considered a measure of migrated cells, which was calculated for percentage of inhibition with respect to control and analyzed using GraphPad prism (6.0) software. 

*Total RNA extraction and cDNA synthesis*

Two treatment doses (1/20 and 1/10 of GI_50_) of AT-101 such as 0.35 μM and 0.7 μM were selected. Control untreated group was also taken and 48 hr of treatment was given to NCI-H522 cell lines, and additionally cells were harvested to extract total RNA and protein. Extraction of RNA was completed through all untreated and treated cell lines using Trizol (Invitrogen) reagent as per instructions given by the manufacturers and stored at -80 °C until additional necessary steps for cDNA synthesis. RNA concentrations and purity were measured using a Thermo Scientific NanoDrop™ 2000 spectrophotometer. 100 ng of total RNA, along with no reverse transcriptase and no DNA controls, was used to synthesize cDNA using Verso (Thermo scientific, USA) following the manufacturer’s protocol. 

*Quantitation of mRNA levels using real-time quantitative PCR (RT-qPCR)*

 20 μl of samples were subjected to RT-qPCR for XIAP, cIAP-1, cIAP-2, Caspase-6, Caspase-7, and β-actin using SYBR Green I technology using the primers indicated in Table 1. XIAP, cIAP-1, cIAP-2, caspase-6, and caspase-7 mRNA expression level study was executed by using the program for 40 cycles, first denaturation step at 94 °C for the 40 sec, annealing was for 20 sec at 60 °C temperature, extension done at 72 °C for 30 sec. The final step for extension was at 72 °C for 10 min. Melting curve analysis was done between the temperature ranges of 35 °C to 90 °C to make sure the target amplification and all the reactions were performed in duplicate. The relative levels of each RNA were determined from the Ct value after normalization with control mRNA and β-actin using a 2-(∆∆CT) (23). Results were shown as mean fold change in cell lines compared with the counterpart. 

*Western blot analysis for IAPs, caspase-6, and caspase-7*

All cells were lysed in lysis buffer, and total protein concentration was estimated by Coomassie Plus Protein Assay Reagent kit (Thermofischer, USA). 30 μg proteins from the cell lysate were separated with sodium dodecyl sulfate-polyacrylamide gel electrophoresis (SDS-PAGE), then transferred to PVDF (polyvinylidene fluoride) membrane film by the semi-dry blotting method (Merck, New Jersey, USA), and probed with respective primary antibodies for 15 hr at 4 °C. Horseradish peroxidase (HRP) secondary antibodies were added and the membrane was stripped after 3 hr at 25 °C incubation. Bands were estimated in ChemiDoc XRS+ by utilizing Image lab programming (Bio-Rad) and standardized with β-actin (1:5000). 

*Computational in silico docking*

Molecular docking protocol was performed as described elsewhere (24). The three-dimensional structures of NFƙB-p50 and XIAP were retrieved from the PDB databank (PDBid: 1svc, 3clx). Bound DNA structure in NFkB-p50 was removed from the pdb file before the docking. The structure was prepared before docking studies using a receptor preparation script from AutoDock-tools. Structures of AT-101 and EC (25) were retrieved from the PubChem database in SDF format ligands which were converted to SYBYL-TRIPOS (mol2) format using BIOVIA-Discovery Studio Visualizer. Converted mol2 files were then modified to AutoDock-VINA format using ligand preparation script from AutoDock tools. The docking box was generated based on the information gained from NFƙB-p50 structure complexed with EC, by selecting two amino acid residues on either side of the active site. Docking was performed by a web tool developed by siBiolead (https://sibiolead.com/) using the AutoDock VINA program. 

*Flow cytometry analysis of NFƙB-p65*

NCI-H522 cells were treated with 3.5 µM or 7.0 µM AT-101 for 48 hr and incubated in a 37 °C/5% CO_2_ incubator. The cells were removed by trypsinization, washed twice with sterile PBS, and re-suspended in HBSS buffer. 0.50 μg/ml FITC- anti-NFƙB-FITC antibody (Thermo Fischer Scientific, Cat #MA5-37157) was added and incubated for 30 min in the dark. After two quick washes with PBS, cells were re-suspended in HBSS buffer. 10,000 events were acquired in a Guava easyCyte™ flow cytometer, and the data were analyzed with InCyte Software from Millipore, (Burlington, CA USA). The mean percentage population of positive cells was presented in comparison with untreated controls.

***Statistical analysis***

All data analysis was performed by using SPSS 20.0 and Graph Pad Prism 6.0 software packages. Relative quantification method 2^-(∆∆CT)^ was used to calculate the mRNA expression of XIAP, cIAP-1, cIAP-2, caspase-6, and caspase-7, and β-actin was used as an internal control. Expressions of XIAP, cIAP-1, cIAP-2, caspase-6, and caspase-7 were presented as mean±SD. While protein band density was observed by the Image Lab software (Bio-Rad). Based on the outcomes parametric t-test, ANOVA, and non-parametric Mann Whitney U and Kruskal Wallis tests were performed to see the statistical differences between the different groups, and *P*-value <0.05 was considered to be statistically significant.

## Results

***AT-101 inhibited lung cancer cell proliferation***

**Figure 1:**
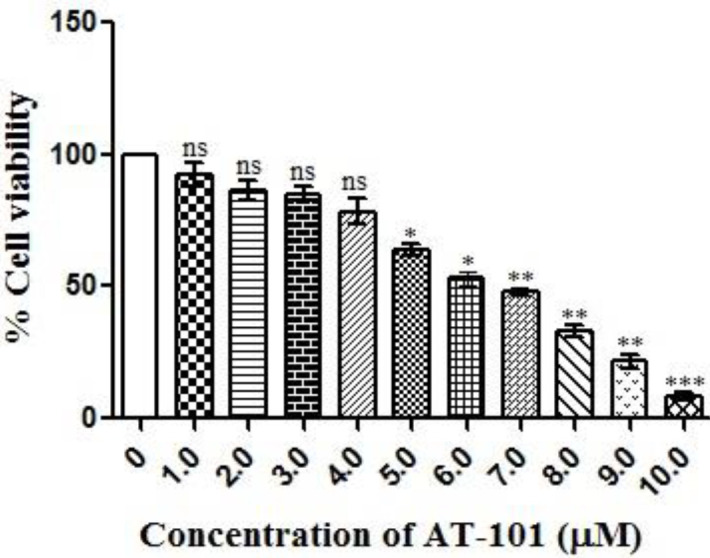
AT101 induces cytotoxic effect in NCI-H522 lung cancer cell line. AT-101 induced cytotoxicity in NCI-H522 cells as confirmed by MTT assay, cell survival was measured with MTT assays with specified concentrations of AT-101 for 48 hr. The results are represented as means ±SD of three independent experiments. Nonsignificant (ns), *P<0.05, **P<0.01, and ***P<0.001 versus untreated control

MTT assay was carried out to analyze the GI_50_ value of AT-101 in the NCI-H522 cell line. Cell proliferation was not inhibited up to 4 μM treatment, while a dose-dependent inhibition of the NCI-H522 cell proliferation was evident from 5 μM to 10 μM concentrations of AT101 (Figure 1). GI_50_ value of AT-101 against NCI-H522 proliferation was calculated to be 7 μM. We used GI_50 and _GI_25 _values for determining functional analysis like cell cycle, apoptosis, and anti-metastasis, while 1/20 and 1/10 of GI_50 _dose (0.35 μM and 0.7 μM) were used for molecular signaling analysis. 

***Effects of AT-101 on NCI-H522 cell function***

**Figure 2:**
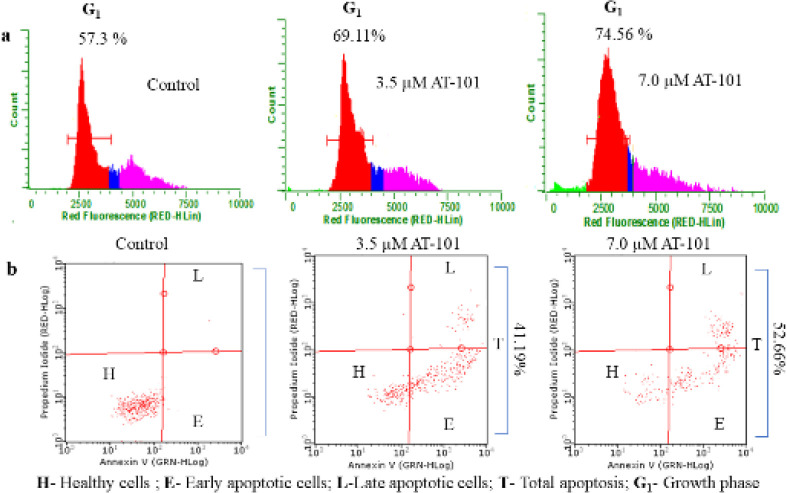
Analysis of cell cycle and apoptotic status of AT-101. (a) Representative histograms from three experiments of AT-101 treated NCI-H522 cells at the end of 24 hr, showing a dose-dependent increase in the G1 phase of the cell cycle. (b) Quadri-plot graphs indicating early and late apoptosis in NCI-H522 cells treated with different concentrations of AT-101 at the end of 48 hr. Numbers indicate the mean total apoptosis percentage from three experiments. Representative graphs are presented

**Figure 3:**
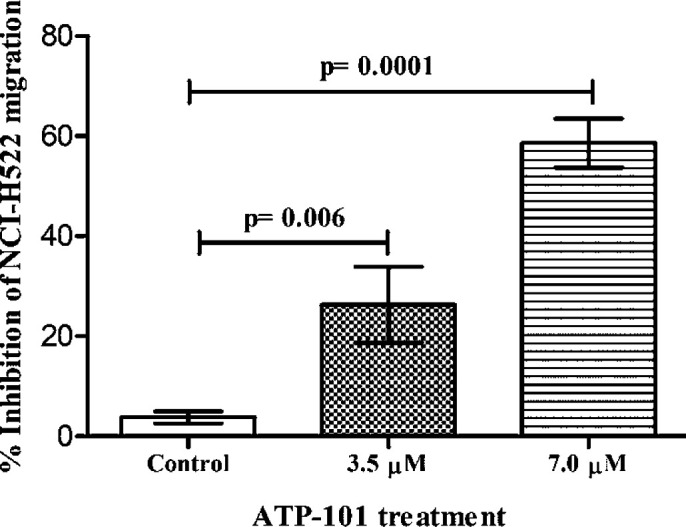
Anti-metastasis property of AT-101. The compound dose-dependently inhibited the transendothelial migration of NCI-H522 cells across HUVEC-membrane inserts under influence of 25 ng/ml HGF as a chemoattractant. The results are represented as means ±SD of three independent experiments. Nonsignificant (ns), *P<0.05, and ***P<0.001 versus untreated control

We first checked whether AT-101 treatment affects the cell cycle regulation of adenocarcinoma cells. The compound dose-dependently arrests NCI-H522 cells in the G_1_ phase of the cell cycle, elevating the corresponding cell population to 69.11% with 3.5 μM treatment from 57.0% of that of untreated control (Figure 2a) at the end of 24 hr. G_1_ arrest was further increased to 74.56% with 7 μM AT-101 treatment (Figure 2a). Next, we checked the effect of AT-101 on the apoptosis level of NCI-H522 cells at 48 hr treatment. The compound was able to dose-dependently increase the early and late phase apoptosis in these cells (Figure 2b). The total apoptosis in these cells were 41.19% and 52.66% with 3.5 μM and 7 μM AT-101 treatments, respectively (Figure 2b). In order to check if there was an anti-metastasis effect of the compound, a tumor cell trans-endothelial cell migration assay was carried out. As indicated in Figure 3, AT-101 dose-dependently inhibited the migration of NCI-H522 cells across HUVEC membrane inserts under the influence of a chemoattractant. 

***AT-101 inhibited the expression*** ***XIAP, cIAP-1, cIAP-2, Caspase-6, and Caspase-7 mRNA expressions***

**Figure 4:**
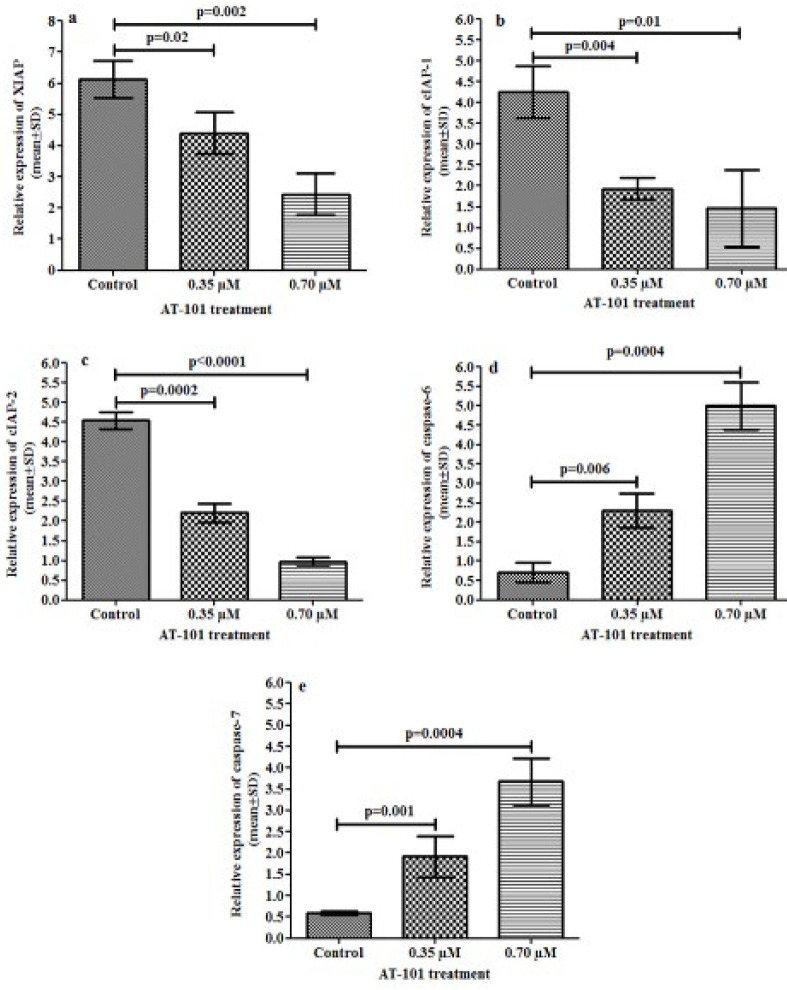
Relative levels of XIAP, cIAP-1, cIAP-2, caspase-6, and caspase-7 mRNA expression in NCI-H522 cell line by RT-qPCR. Levels of mRNA encoding (a) XIAP (b), cIAP-1, (c) cIAP-2, (d) caspase-6, and (e) caspase-7 show the significant difference in the levels of each mRNA after treating with 0.35 and 0.70 μM of AT-101 in comparison with the untreated controls. After normalizing to actin, results from three individual experiments were expressed as mean ± SD and plotted

Treatment of AT-101 showed decreased XIAP, cIAP-1, and cIAP-2 mRNA expressions compared with the untreated control group (Figure 4). It was observed that untreated control cells showed 6.13±0.59-fold XIAP mRNA expression, while treatment with 0.35 μM and 0.7 μM decreased XIAP mRNA expression significantly (1.93±0.26, 2.44±0.67 folds, *P*=0.02 and *P*=0.002, respectively) (Figure 4a). Similarly, the relative levels of cIAP-1 mRNAs in the untreated control group showed 4.24±0.62 fold, while AT-101 treatment significantly reduced cIAP-1 mRNAs (1.93±0.26-fold *P*=0.004 when treated with 0.35 μM and 1.46±0.93 *P*=0.01 with 0.7 μM) (Figure 4b). Likewise, cIAP-2 mRNA expression was depleted when treated with 0.35 μM of AT-101 for 2.20±0.24-fold (*P*=0.0002), and further 0.97±0.10-fold (*P*<0.0001) with 0.7 μM of AT101(Figure 4c). 

However, AT-101 showed an increase in caspase-6 and caspase-7 mRNA expressions in comparison with the untreated controls. The level of caspase-6 mRNA in the untreated control group was 0.71±0.26-fold, while treatment with 0.35 μM and 0.7 μM increased the expression of caspase-6 mRNA significantly as illustrated in Figure 4d (2.30±0.44-fold; *P*=0.006 and 5.0±0.60-fold; *P*=0.0004, respectively). An increase of caspase-7 mRNA expression was observed in treated groups (Figure 4e), where the untreated control group showed 0.50±0.04-fold caspase-7 mRNA expression while treatment with 0.35 μM and 0.7 μM showed 1.92±0.48-fold and 3.67±0.53-fold caspase-7 mRNA expression, respectively (*P*=0.008 and *P* =0.0006).    

***AT-101 treatment*** ***and XIAP, cIAP-1, cIAP-2, Caspase-6, and Caspase-7 protein expression***

**Figure 5:**
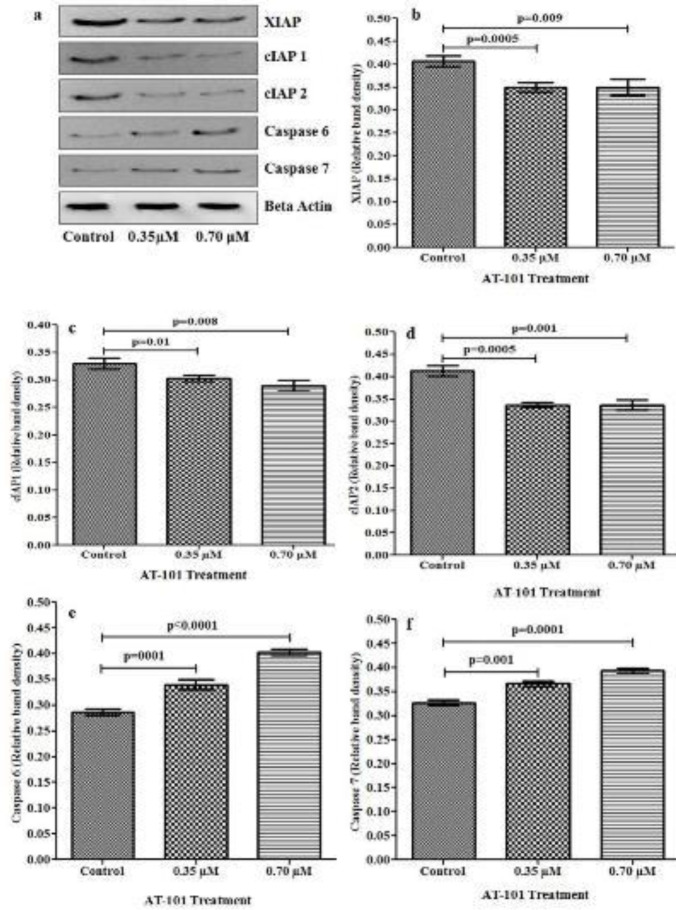
Levels of XIAP, cIAP-1, cIAP-2, caspase-6, and caspase-7 protein expression in NCI-H522 by western blotting. (a) Western blot of anti-apoptotic markers after various doses of AT-101 treatment. Levels of (b) XIAP (c), cIAP-1, (d) cIAP-2, (e) caspase-6, and (f) caspase-7 proteins show the significant difference in the levels of each protein after treating with 0.35 and 0.70 μM of AT-101 in comparison with the untreated control. After normalizing to actin, results from three individual experiments were expressed as mean ± SD and plotted

In order to confirm that observed mRNA alterations are translated in the protein expressions, we performed western blotting for the same parameters. Lung cancer cell line NCI-H522 treated with AT-101 showed a significant decrease in XIAP, cIAP-1, and cIAP-2 protein expressions compared with the control group (Figure 5a). AT-101 treatment with 0.35 μM and 0.7 μM showed a significant reduction in XIAP protein expression compared with untreated controls (*P*=0.0005 and *P*=0.009 respectively) (Figure 5b). It was also observed that the expression of the cIAP-1 protein in treatment with 0.35 μM and 0.7 μM of AT-101 was significantly reduced when compared with untreated controls (*P*=0.01 and *P*=0.008, respectively) (Figure 5c). Decreased protein expression of cIAP-2 was observed when treating with 0.35 μM and 0.7 μM of AT101 in comparison with untreated controls (*P*=0.0005 and *P*=0.001, respectively) (Figure 5 d). 

**Figure 6:**
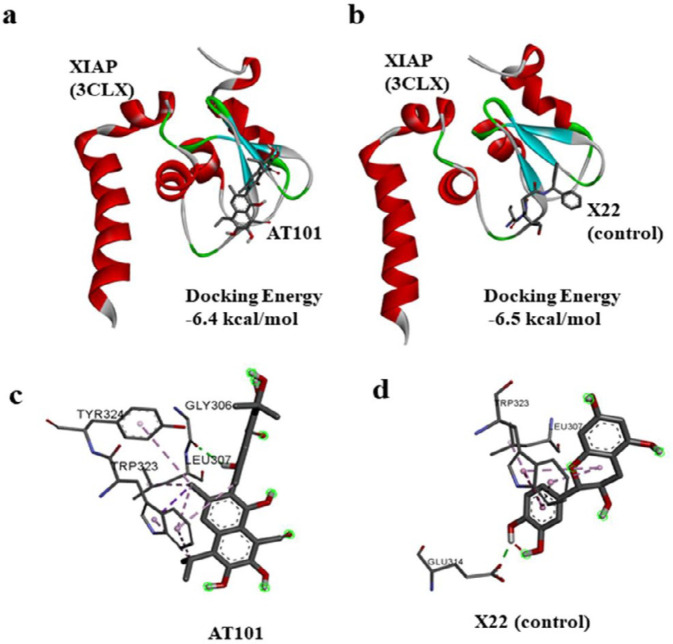
Affinity and inhibition analysis of XIAP with AT-101. (a) In silico docking prediction of AT-101 with XIAP (b) X22 was used as a positive control to predict docking position, area as indicated by the inner circle. (c) XIAP interacting residues with AT101. (d) XIAP interacting residues with control

In contrast to that, AT-101 treatment showed increased protein expression of caspase-6 and caspase - 7 levels in NCI H522 cell lines when compared with the untreated controls (Figures 5 e, f). Caspase-6 protein levels were significantly elevated with 0.35 μM and 0.7 μM AT-101 treatments compared with untreated controls (*P*=0.0001 and *P*<0.0001, respectively). Increased level of caspase-7 protein was also observed with treatments of 0.35 μM and 0.7 μM compared with untreated controls (*P*=0.001 and *P*=0.0001, respectively). Studies have previously shown that SMAC derivatives bind directly to XIAP (26). Since AT-101 is also a SMAC derivative, we predicted the binding modes of AT-101 with XIAP (27). For docking analysis, we used the SMAC005 bound XIAP complex. Results show AT-101 binds XIAP efficiently (-6.4 kcal/mol) similar to SMAC005 (positive control) (Figures 6 a, b). 

***Interaction of AT-101 with NFƙB in lung adenocarcinoma cells***

**Figure 7:**
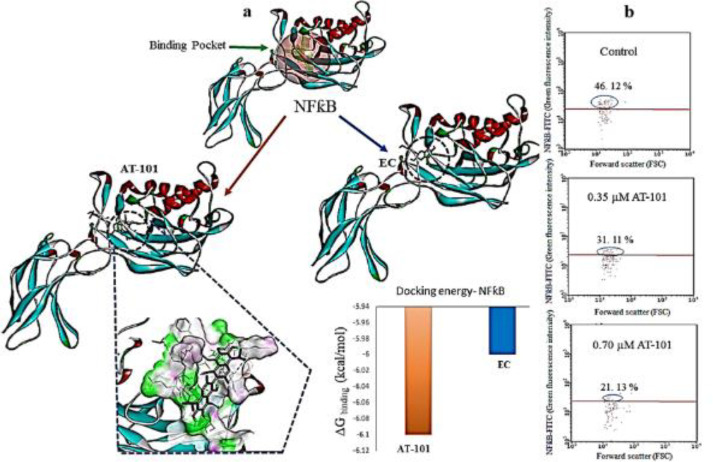
Affinity and inhibition analysis of NFƙB with AT-101. (a) In silico docking predication of AT-101 with NFƙB. -epicatechin (EC) was used as a positive control to predict docking position, area as indicated by the inner circle. (b) Representative flow cytometry graphs of NFƙB in NCI-H522 cells with or without AT-101 treatment at end of 48 hr. Numbers are mean NFƙB positive populations from three different experiments

We then performed a docking analysis with the compound to NFƙB. To check the proper docking position, we used a known NFƙB-inhibitor, -epicatechin (EC) (26). As indicated in Figure 6, AT-101 bound to the predicted binding area of Epicatechin. However, when analyzed for the binding affinity energy (ΔG binding) level, AT-101 was predicted even better than EC with -6.10 kcal/mol, while EC prediction was -6.0 kcal/mol (Figure 6a). PLIP (Protein-Ligand Interaction Profiler) analysis revealed critical interacting residues to be LYS-147, GLU-207, and SER-211 (Figure 7a). Since the activation of NF-KB requires a heterodimer complex between p50/p65 (28), blocking p50 could impede heterodimer complex formation between p50/p65. Further confirmation of this prediction was carried out by flow cytometry analysis. The untreated NCI-H522 cells have 46.12% NFƙB positive population, which dose-dependently decreased with AT-101 treatments of 0.35 µM and 0.7 µM (Figure 7b). 

**Table 1:** Sequence of primers used in real-time quantitative PCR

**Gene **	**Primers sequences (5'-3')**
**XIAP**	GCACGAGCAGGGTTTCTTTATACTGGTG (F)
	CTTCTTCACAATACATGGCAGGGTTCCTC (R)
**cIAP1**	GAATACTCCCTGTGATTAATGGTGCCGTGG (F)
	TCTCTTGCTTGTAAAGACGTCTGTGTCTTC (R)
**cIAP2**	GGGCAGCAGGTTTACAAAGG (F)
	TCCCTTTAAGGATTTTAGGTCTCCA (R)
**Caspase6**	TACAAAATGGACCACAGGAGGAG (F)
	ACACAAAGCAATCGGCATCT (R)
**Caspase7**	ATGGCAGATGATCAGGGCTGT (F)
	CTATTGACTGAAGTAGAGTTCC (R)
**Beta actin**	CGCAAAGACCTGTACGCCAAC (F)
	GAGCCGCCGATCCACACG (R)

## Discussion

Disease recurrence and progression owing to apoptosis-machinery failure and chemotherapeutic resistance due to increased expression of IAPs has been a major issue in cancer management (29). Apoptosis is a form of programmed cell death that appears under natural physiological situations and is defined as being either extrinsic (death receptor-dependent) or intrinsic (mitochondria-dependent) (30). Where the programmed cell death fails, it allows the cancer cells to mutate, grow, and proliferate. Hence, reenergizing their capability towards apoptosis is an interesting anti-cancer therapy approach and has been investigated in a variety of chemotherapeutic drug strategies (30). Antagonists of IAPs, such as the second mitochondria-derived activator of caspases (SMAC/DIABLO) bind to the BIR domains of IAPs to inhibit them (31). SMAC prevents XIAP from binding as well as inhibiting caspases and promoting cIAPs auto-ubiquitylation and proteasomal degradation (32). Though IAPs employ their anti-apoptotic programs through the direct inhibitory effect of initiator and effector caspases, they have also been exhibited to ubiquitinate caspase proteins, thus indirectly inhibit apoptosis (11). 

Results from this study indicated an increase in G_1_ phase arrest of NCI-H522 cells with AT-101 treatment. In general, a tight regulation exists between cell proliferation/death in order to maintain tissue homeostasis. Several studies suggest that this regulation can be achieved by linking cell cycle progression and programmed cell death that may be driven by common factors (34). Therefore, an argument of a link between the cell cycle and apoptosis arises in favor of a stall in cell cycle arrest that may further induce an apoptotic response (33). Our observations also indicated an increase in apoptosis level at the end of 48 hr AT-101 treatment, while G_1_ cell cycle arrest was observed at 24 hr post AT-101 treatments. Next, when checked for anti-metastatic effects if any, results demonstrated a clear dose-dependent inhibition of tumor cell migration of NCI-H522 cells across the HUVEC membrane. These observations were on par with the established anti-metastasis effects by other SMAC mimetic compounds (34). 

SMAC mimetic has been established to focus multiple IAPs, comprising cIAP1, c-IAP2, and XIAP (35). The present study observed that AT-101 treatment to NCI-H522 cell line with 2 different doses, such as 0.35 μM and 0.7 μM, concentration has an impact on the down-regulation of XIAP, cIAP1, and cIAP2 mRNA expression while untreated cells had high expression of XIAP, cIAP1, and cIAP2 mRNA. An increase in caspase-6 and caspase-7 mRNA expression levels was observed in AT-101 treated cells while untreated control cells showed low caspase-6 and caspase 7 mRNA expressions. XIAP overexpression in cell lines and cancer tissues has been revealed to inhibit apoptosis stimulated by a variety of apoptotic stimuli, including Fas, tumor necrosis factor, withdrawal of serum or growth factor, and radiation therapy (36). A current association between poor prognosis and elevated XIAP along with short existence has been established in patients with severe myelogenous leukemia (11). It is reported that XIAP is extremely overexpressed in several cancer cell lines of the NCI (NIH) panel (37). A study by Tong *et al*. showed that down-regulation of XIAP could induce apoptosis and enhance chemotherapy sensitivity in gastric cancer cells (38). Similarly, we observed that AT-101 treatment showed down-regulation of XIAP, cIAP1, and cIAP2 protein expression that led toward increased expression of caspase-6 and caspase-7 protein expression compared with its counterpart. A study by Hu *et al*. showed that SMAC mimetic AT-101 was significantly involved in induction of apoptosis in cisplatin-resistant ovarian cancer cells and helped to understand the improved treatment outcome in human ovarian cancer (39).

It was proven that XIAP played a fundamental role in the resistance of anoikis and cancer metastasis in prostate cancer (40). Many pieces of research disclosed that a high level of XIAP in cancer patients expected poor diagnosis or low existence rates. Currently, XIAP has been recommended as an appealing target for the latest anti-tumor interventions. A compound embelin, extracted from Embelia Ribes’s fruits, was described as an essential XIAP inhibitor used by the practitioner (41). Second mitochondrial activator of caspases (SMAC) mimetics has been exhibited to reduce the growth of the tumor cells with slight toxicity to the normal cells (42). Presently, the therapeutic value of targeting XIAP in regulating the development of cancer and improving the sensitivity of chemotherapy has been testified in many types of cancer (43).

We next asked if the efficacy of AT-101 to induce IAPs/ caspase mediated apoptosis had an influence on NFκB in this response. Therefore, AT-101 was computationally docked to NFκB-p50 protein. AT-101’s affinity towards NFκB-p50 was confirmed from our *in silico* docking and flow cytometry analysis. The binding of AT101 with p50 may inhibit the heterodimer formation between p50/p65. Since heterodimer formation between p50/p65 is a crucial step in the activation and nuclear translocation of NF-KB, inhibiting p50 would impede its downstream steps (28). This was again confirmed by the flow cytometry results of reduction in the NFκB-p65 levels when treated with AT-101. It has been shown that cIAP1 and cIAP2 moderate their anti-apoptotic action through NF-kB signaling mediated by TNF-α (44). Another study indicated intermolecular cooperation between IAP proteins, XIAP, and survivin to promote tumor cell invasion and metastasis centrally driven by NFκB activation (45). The same study also indicated that NFκB-dependent gene expression occurs upstream in IAP-mediated metastasis (45). In addition to the cytoprotective effects, IAPs have also shown to exhibit a broader regulation of cellular homeostasis through NFκB (46). This evidence favors that a cross-talk exists between IAPs and NFκB in tumor cell regulation and homeostasis. Therefore, it can be inferred that the observed anti-proliferative, anti-metastatic, and apoptosis induction efficacy of AT-101 in NCI-H522 cells could be mediated by p65-NFƙB mediated IAPs/ caspase regulation. However, further research on the detailed pathway including more *in vitro* and *in vivo* investigations are recommended as future directions to this study. 

## Conclusion

The present study revealed that the SMAC mimetic compound AT101 treatment down-regulated XIAP, cIAP1, and cIAP2 mRNA and protein expressions while increasing caspase-6 and caspase-7 mRNA and protein expression levels in NCI-H522 lung cancer cell line to favor total apoptosis in these cells. The compound also exhibited anti-metastatic effects and decreased NFƙB-p65 in the lung adenocarcinoma cells. Data suggested that SMAC mimetic AT-101 could be used as a potential new therapeutic approach in lung cancer management.

## References

[1] Siegel R, Naishadham D, Jemal A (2013). Cancer statistics, 2013. CA Cancer J Clin.

[2] Chen PL, Zhao T, Feng R, Chai J, Tong GX, Wang DB (2014). Patterns and trends with cancer incidence and mortality rates reported by the China National Cancer Registry. Asian Pac J Cancer Prev.

[3] Kroemer G, Levine B (2008). Autophagic cell death: the story of a misnomer. Nat Rev Mol Cell Biol.

[4] Tsujimoto Y, Shimizu S (2005). Another way to die: autophagic programmed cell death. Cell Death Differ.

[5] Eckelman BP, Salvesen GS, Scott FL (2006). Human inhibitor of apoptosis proteins: why XIAP is the black sheep of the family. EMBO Rep.

[6] Scott FL, Denault JB, Riedl SJ, Shin H, Renatus M, Salvesen GS (2005). XIAP inhibits caspase-3 and -7 using two binding sites: evolutionarily conserved mechanism of IAPs. Embo J.

[7] Obexer P, Ausserlechner MJ (2014). X-Linked inhibitor of apoptosis protein – a critical death resistance regulator and therapeutic target for personalized cancer therapy. Front Oncol.

[8] Kim JY, Morgan M, Kim DG, Lee JY, Bai L, Lin Y (2011). TNFα induced noncanonical NF-κB activation is attenuated by RIP1 through stabilization of TRAF2. J Cell Sci.

[9] Vince JE, Pantaki D, Feltham R, Mace PD, Cordier SM, Schmukle AC (2009). TRAF2 must bind to cellular inhibitors of apoptosis for tumor necrosis factor (tnf) to efficiently activate nf-{kappa}b and to prevent tnf-induced apoptosis. J Biol Chem.

[10] Mansouri A, Zhang Q, Ridgway LD, Tian L, Claret FX (2003). Cisplatin resistance in an ovarian carcinoma is associated with a defect in programmed cell death control through XIAP regulation. Oncol Res.

[11] Tamm I, Kornblau SM, Segall H, Krajewski S, Welsh K, Kitada S (2000). Expression and prognostic significance of IAP-family genes in human cancers and myeloid leukemias. Clin Cancer Res.

[12] Akyurek N, Ren Y, Rassidakis GZ, Schlette EJ, Medeiros LJ (2006). Expression of inhibitor of apoptosis proteins in B-cell non-Hodgkin and Hodgkin lymphomas. Cancer.

[13] Bilim V, Yuuki K, Itoi T, Muto A, Kato T, Nagaoka A (2008). Double inhibition of XIAP and Bcl-2 axis is beneficial for retrieving sensitivity of renal cell cancer to apoptosis. Br J Cancer.

[14] Dizdar L, Jünemann LM, Werner TA, Verde PE, Baldus SE, Stoecklein NH (2018). Clinicopathological and functional implications of the inhibitor of apoptosis proteins survivin and XIAP in esophageal cancer. Oncol Lett.

[15] Yan Y, Mahotka C, Heikaus S, Shibata T, Wethkamp N, Liebmann J (2004). Disturbed balance of expression between XIAP and Smac/DIABLO during tumour progression in renal cell carcinomas. Br J Cancer.

[16] Yan Y, Mahotka C, Heikaus S, Shibata T, Wethkamp N, Liebmann J (2004). Disturbed balance of expression between XIAP and Smac/DIABLO during tumour progression in renal cell carcinomas. Br J Cancer.

[17] Obexer P, Ausserlechner MJ (2014). X-linked inhibitor of apoptosis protein - a critical death resistance regulator and therapeutic target for personalized cancer therapy. Front Oncol.

[18] Abbas R, Larisch S (2020). Targeting XIAP for promoting cancer cell death—the story of ARTS and. SMAC..

[19] Shi Y (2001). A structural view of mitochondria-mediated apoptosis. Nat Struct Biol.

[20] Fulda S, Vucic D (2012). Targeting IAP proteins for therapeutic intervention in cancer. Nat Rev Drug Discov.

[21] Meng Y, Tang W, Dai Y, Wu X, Liu M, Ji Q (2008). Natural BH3 mimetic (-)-gossypol chemosensitizes human prostate cancer via Bcl-xL inhibition accompanied by increase of Puma and Noxa. Mol Cancer Ther.

[22] Prasanna R, Harish CC (2010). Anticancer effect of a novel 2-arylidene-4,7-dimethyl indan-1-one against human breast adenocarcinoma cell line by G2/M cell cycle arrest. Oncol Res.

[23] Livak KJ, Schmittgen TD (2001). Analysis of relative gene expression data using real-time quantitative PCR and the 2(-Delta Delta C(T)) Method. Methods.

[24] Mukund V, Behera SK, Alam A, Nagaraju GP (2019). Molecular docking analysis of nuclear factor-κB and genistein interaction in the context of breast cancer. Bioinformation.

[25] Suhail M PA, Husain A, Rehan M (2019). Exploring inhibitory mechanisms of green tea catechins as inhibitors of a cancer therapeutic target, nuclear factor-κB (NF-κB). Biosci Biotech Res Asia.

[26] Mastrangelo E, Cossu F, Milani M, Sorrentino G, Lecis D, Delia D (2008). Targeting the X-linked inhibitor of apoptosis protein through 4-substituted azabicyclo[5 3 0]alkane smac mimetics Structure activity and recognition principles. J Mol Biol.

[27] Nejabat M, Soltani F, Alibolandi M, Nejabat M, Abnous K, Hadizadeh F (2020). Smac peptide and doxorubicin-encapsulated nanoparticles: design, preparation, computational molecular approach and in vitro studies on cancer cells. J Biomol Struct Dyn.

[28] Giridharan S, Srinivasan M (2018). Mechanisms of NF-κB p65 and strategies for therapeutic manipulation. J Inflamm Res.

[29] Galluzzi L, Vitale I, Aaronson SA, Abrams JM, Adam D, Agostinis P (2018). Molecular mechanisms of cell death: recommendations of the Nomenclature Committee on Cell Death 2018. Cell Death Differ.

[30] Salvesen GS, Duckett CS (2002). IAP proteins: blocking the road to death’s door. Nat Rev Mol Cell Biol.

[31] Silke J, Hawkins CJ, Ekert PG, Chew J, Day CL, Pakusch M (2002). The anti-apoptotic activity of XIAP is retained upon mutation of both the caspase-3 and caspase-9 interacting sites. J Cell Biol.

[32] Flygare JA, Beresini M, Budha N, Chan H, Chan IT, Cheeti S (2012). Discovery of a potent small-molecule antagonist of inhibitor of apoptosis (IAP) proteins and clinical candidate for the treatment of cancer (GDC-0152). J Med Chem.

[33] Pucci B, Kasten M, Giordano A (2000). Cell cycle and apoptosis. Neoplasia.

[34] Shekhar TM, Burvenich IJG, Harris MA, Rigopoulos A, Zanker D, Spurling A (2019). Smac mimetics LCL161 and GDC-0152 inhibit osteosarcoma growth and metastasis in mice. BMC Cancer.

[35] Martinez-Ruiz G, Maldonado V, Ceballos-Cancino G, Grajeda JP, Melendez-Zajgla J (2008). Role of Smac/DIABLO in cancer progression. J Exp Clin Cancer Res.

[36] Notarbartolo M, Cervello M, Poma P, Dusonchet L, Meli M, D’Alessandro N (2004). Expression of the IAPs in multidrug resistant tumor cells. Oncol Rep.

[37] Fong WG, Liston P, Rajcan-Separovic E St, Jean M, Craig C, Korneluk RG (2000). Expression and genetic analysis of XIAP-associated factor 1 (XAF1) in cancer cell lines. Genomics.

[38] Tong QS, Zheng LD, Wang L, Zeng FQ, Chen FM, Dong JH (2005). Downregulation of XIAP expression induces apoptosis and enhances chemotherapeutic sensitivity in human gastric cancer cells. Cancer Gene Ther.

[39] Hu W, Wang F, Tang J, Liu X, Yuan Z, Nie C (2012). Proapoptotic protein Smac mediates apoptosis in cisplatin-resistant ovarian cancer cells when treated with the anti-tumor agent AT101. J Biol Chem.

[40] Berezovskaya O, Schimmer AD, Glinskii AB, Pinilla C, Hoffman RM, Reed JC (2005). Increased expression of apoptosis inhibitor protein XIAP contributes to anoikis resistance of circulating human prostate cancer metastasis precursor cells. Cancer Res.

[41] Lu H, Wang J, Wang Y, Qiao L, Zhou Y (2016). Embelin and its role in chronic diseases. Adv Exp Med Biol.

[42] Fulda S (2015). Promises and challenges of smac mimetics as cancer therapeutics. clin cancer res.

[43] Prabhu KS, Achkar IW, Kuttikrishnan S, Akhtar S, Khan AQ, Siveen KS (2018). Embelin: A benzoquinone possesses therapeutic potential for the treatment of human cancer. Future Med Chem.

[44] LaCasse EC, Mahoney DJ, Cheung HH, Plenchette S, Baird S, Korneluk RG (2008). IAP-targeted therapies for cancer. Oncogene.

[45] Mehrotra S, Languino LR, Raskett CM, Mercurio AM, Dohi T, Altieri DC (2010). IAP regulation of metastasis. Cancer Cell.

[46] Srinivasula SM, Ashwell JD (2008). IAPs: what’s in a name?. Mol Cell.

